# Exploring circular MET RNA as a potential biomarker in tumors exhibiting high MET activity

**DOI:** 10.1186/s13046-023-02690-5

**Published:** 2023-05-12

**Authors:** Francesca Bersani, Francesca Picca, Deborah Morena, Luisella Righi, Francesca Napoli, Mariangela Russo, Daniele Oddo, Giuseppe Rospo, Carola Negrino, Barbara Castella, Marco Volante, Angela Listì, Vanessa Zambelli, Federica Benso, Fabrizio Tabbò, Paolo Bironzo, Emanuele Monteleone, Valeria Poli, Filippo Pietrantonio, Federica Di Nicolantonio, Alberto Bardelli, Carola Ponzetto, Silvia Novello, Giorgio V. Scagliotti, Riccardo Taulli

**Affiliations:** 1 Department of Oncology, University of Torino, Orbassano, Italy; 2 Center for Experimental Research and Medical Studies (CeRMS), AOU Città della Salute e della Scienza di Torino, Turin, Italy; 3 Pathology Unit, Department of Oncology at San Luigi Hospital, University of Torino, Orbassano, Italy; 4 Candiolo Cancer Institute, FPO-IRCCS, Candiolo, Italy; 5 Laboratorio di Immunologia dei Tumori del Sangue (LITS), Centro Interdipartimentale di Ricerca in Biologia Molecolare (CIRBM), University of Torino, Turin, Italy; 6 Thoracic Unit and Medical Oncology Division, Department of Oncology at San Luigi Hospital, University of Torino, Orbassano, Italy; 7 Department of Molecular Biotechnology and Health Sciences, University of Torino, Turin, Italy; 8 Università Vita-Salute San Raffaele, Milan, Italy; 9 Medical Oncology Department, Fondazione IRCCS Istituto Nazionale Dei Tumori, Milan, Italy; 10 Department of Oncology and Hemato-Oncology, University of Milano, Milan, Italy; 11 IFOM, Istituto Fondazione di Oncologia Molecolare ETS, Milan, Italy

**Keywords:** Circular RNA, Biomarker, Receptor tyrosine kinase, Molecular-targeted therapy resistance

## Abstract

**Background:**

MET-driven acquired resistance is emerging with unanticipated frequency in patients relapsing upon molecular therapy treatments. However, the determination of *MET* amplification remains challenging using both standard and next-generation sequencing-based methodologies. Liquid biopsy is an effective, non-invasive approach to define cancer genomic profiles, track tumor evolution over time, monitor treatment response and detect molecular resistance in advance. Circular RNAs (circRNAs), a family of RNA molecules that originate from a process of back-splicing, are attracting growing interest as potential novel biomarkers for their stability in body fluids.

**Methods:**

We identified a circRNA encoded by the *MET* gene (circMET) and exploited blood-derived cell-free RNA (cfRNA) and matched tumor tissues to identify, stratify and monitor advanced cancer patients molecularly characterized by high MET activity, generally associated with genomic amplification.

**Results:**

Using publicly available bioinformatic tools, we discovered that the *MET* locus transcribes several circRNA molecules, but only one candidate, circMET, was particularly abundant. Deeper molecular analysis revealed that circMET levels positively correlated with MET expression and activity, especially in *MET*-amplified cells. We developed a circMET-detection strategy and, in parallel, we performed standard FISH and IHC analyses in the same specimens to assess whether circMET quantification could identify patients displaying high MET activity. Longitudinal monitoring of circMET levels in the plasma of selected patients revealed the early emergence of *MET* amplification as a mechanism of acquired resistance to molecular therapies.

**Conclusions:**

We found that measurement of circMET levels allows identification and tracking of patients characterized by high MET activity. Circulating circMET (ccMET) detection and analysis could be a simple, cost-effective, non-invasive approach to better implement patient stratification based on MET expression, as well as to dynamically monitor over time both therapy response and clonal evolution during treatment.

**Supplementary Information:**

The online version contains supplementary material available at 10.1186/s13046-023-02690-5.

## Background

Circular RNAs (circRNAs) are a dynamic, evolutionarily conserved class of stable RNA molecules whose biological functions are largely unknown. Originally discovered in early 1990s [1], circRNAs were naively considered as a rare phenomenon in biology. Recent advances in next generation sequencing (NGS) technologies and the development of novel bioinformatic pipelines have twisted this concept [2–4]. CircRNA biogenesis has been extensively investigated and reviewed [5–8]. Generally, circRNAs are produced by a process called back-splicing in which the downstream 3’ splice site of an exon is covalently joined to the upstream 5’ splice site, forming a circular RNA molecule with a 3’-5’ phosphodiester bond at the back-splicing junction (BSJ) site. Alternatively, the failure of intron lariat debranching results in another subtype of circRNAs characterized by a 2’-5’ phosphodiester bond [9, 10]. This process is modulated by intronic complementary sequences (ICSs), mainly Alu sequences in primates, localized in the flanking introns of circularized exons [11, 12] and it is facilitated or inhibited by specific RNA binding proteins [13, 14]. Typically, back-splicing is much less productive than canonical linear splicing [15], thus the rate of linear and circRNA species is significantly different [16, 17]. On the other hand, circularization confers intrinsic resistance to exonuclease [2], resulting in higher stability and prolonged half-life compared to the cognate linear RNA [15, 18]. Remarkably, circRNA abundance is regulated in a cell- or tissue-specific manner and it changes significantly in specific biological and pathological conditions, including cancer [19–22]. Moreover, altered expression of circRNAs has been successfully detected in different body fluids (such as blood, plasma, saliva and urine) of patients affected by a variety of diseases, including cancer, suggesting the potential clinical applications of circRNAs as innovative biomarkers [17, 19, 23–30].

Liquid biopsy stands out as an effective and minimally invasive approach to comprehensively annotate the genomic profile of solid tumors directly from the blood. This strategy is also extremely effective to monitor minimal residual disease and for early prediction of tumor recurrence [31]. This last application is particularly relevant at the therapeutic level, since a precocious detection of actionable targets responsible for clinical relapse could inform and guide additional lines of treatment, thus improving patient benefit and outcome. The vast majority of studies exploit the potential of circulating tumor DNA (ctDNA) analysis, which represents the ideal setting for the detection of cancer-related mutations and other aberrant genomic alterations. Although mRNA detection in the plasma was initially observed in 1996 [32] and blood-based comprehensive RNA tumor profiling was shown to be highly informative [33, 34], the full potential of cell-free RNA (cfRNA) is still under active investigation and its clinical implications remain unclear.

MET is the transmembrane tyrosine kinase receptor for hepatocyte growth factor/scatter factor (HGF/SF) [35]. In physiological conditions, MET signaling is activated upon HGF binding to the extracellular domain of the receptor [36]. Physiological HGF/MET signaling is relevant for mammalian development and tissue regeneration, although the same pathway is frequently dysregulated in cancer [37]. Notably, *MET* overexpression and amplification, rather than activating point mutations, have emerged as key mechanisms of acquired resistance to anti-EGFR and anti-ALK treatments [38–40]. To date, the identification of patients who could benefit from anti-MET therapies remains extremely challenging, since standard diagnostic assays (i.e. IHC and FISH) and amplicon-based next-generation sequencing (NGS) approaches present intrinsic technical limitations. Even the definition of unambiguous criteria to classify patients displaying high MET activity to guide treatment-related decisions currently remains an unmet need, since both more traditional IHC or FISH analyses and advanced NGS technologies have not defined univocal cutoffs [41, 42].

Here we provide a comprehensive molecular characterization of circular MET RNA (circMET), an abundant and stable circRNA molecule encoded by *MET* exon 2. Moreover, we establish a circMET-based molecular annotation to verify that circMET expression reflects the status of MET activity in both cell lines and tumor specimens. Finally, we demonstrate that circulating circMET (ccMET) quantitation could represent a simple and minimally invasive strategy to detect and to monitor MET-driven acquired resistance directly in the plasma of oncologic patients.

## Methods

### Cell culture and treatments

All cells were incubated at 37 °C in a 5% CO_2_-water-saturated atmosphere and cultured in DMEM or RPMI media (Sigma-Aldrich) supplemented with 10% fetal bovine serum (GIBCO), 2 mM L-glutamine, 100 U/ml penicillin and 0.1 mg/ml streptomycin (Sigma-Aldrich). Cell lines were routinely authenticated by short tandem repeat (STR) analysis, and regularly tested with MycoAlert (Lonza) to ascertain the absence of any mycoplasma infection. #1640 HGF-overexpressing primary murine sarcoma cells [43] and resistant WiDr cells [44] were previously described. HCC827 resistant cells were obtained by prolonged treatment of parental cells, or their single cell-derived sub clones, with cetuximab (50 µg/ml), afatinib (0.05 µM) or erlotinib (0.5 µM). Moreover, the selected cl. 37 and cl. 39 AFAT res. HCC827 cell line were treated with crizotinib (1 µM) alone or in combination with afatinib (0.05 µM). All drugs were purchased from Selleckchem. Actinomycin D (Sigma-Aldrich) was used at 10 µg/ml for the indicated time spans.

### Tumor samples and plasma collection

We retrospectively analyzed histologically confirmed lung adenocarcinomas from S. Luigi Gonzaga Hospital (Orbassano, Italy) and AOU Città della Salute e della Scienza di Torino Hospital (Torino, Italy) and colon carcinomas from Fondazione IRCCS Istituto Nazionale dei Tumori (Milano, Italy). Patient samples evaluated in this study were selected based on the *MET* status [IHC for MET and pMET and/or FISH-derived *MET* GCN or *MET*/chromosome enumeration probe 7 (CEP7) ratio] determined for clinical purposes and considering the availability of tumor tissues. Tumor specimens were classified as ‘High/Low MET’ based on pMET positivity by IHC, indicating a functionally active MET protein, or, in the few cases where this information was missing, on strong *MET* GCN amplification identified by FISH. All tumor samples were formalin fixed paraffin-embedded (FFPE). *EGFR* status in tumor samples was assessed as described previously [45, 46]. CT scans were obtained as part of routine clinical care. Whole blood was collected by blood draw using EDTA as anticoagulant. Plasma was separated within 5 h through 2 different centrifugation steps (the first at room temperature for 10 min at 1600 g and the second at 3000 g for the same time and temperature). Plasma was stored at − 80 °C until DNA/RNA extraction. Mouse rhabdomyosarcoma specimens included in circMET analysis were described previously [43].

### Primer design, PCR and Sanger sequencing

Two types of circMET-specific divergent primers (specified in Table S4) were designed: non-junction-spanning primers allowed detection of human exon 2 (or mouse exon 3)—containing MET circular RNA. In contrast, junction-spanning (JS) primers were specific for circMET detection since the sense oligonucleotide spanned the hsa_circ_0082002 back-spliced junction. Genomic DNA or cDNA were PCR amplified using the GoTaq G2 Flexi DNA Polymerase (Promega) according to the manufacturer’s instructions. Thermal cycling conditions were the following: 94 °C for 4 min, then 35 cycles of 94 °C for 20 s, 56 °C for 45 s, and 72 °C for 45 s, followed by a final step at 72 °C for 5 min. A no template control (NTC) was included in every assay. PCR products were run on 3% agarose gels. For Sanger sequence of circMET back-spliced junction, PCR was performed with divergent non-junction-spanning primers. The band corresponding to circMET was gel extracted using QIAquick Gel Extraction Kit (QIAGEN) and Sanger sequenced with the same non-junction-spanning divergent primers used for PCR amplification.

### Total RNA extraction

Total RNA from cell lines was extracted using TRI Reagent Solution (Thermo Fisher Scientific) or Direct-zol RNA Miniprep (Zymo Research) according to the manufacturer’s instructions. Total RNA from FFPE samples was extracted from 5 sections of 5 µm thickness using High Pure FFPET RNA isolation kit (Roche) according to the manufacturer’s instructions. Total RNA was purified from plasma using two extraction kits, according to the available volume of plasma samples. MiRNeasy Serum/Plasma Advanced Kit (QIAGEN) was used, according to the manufacturer’s instructions, to extract RNA from 600 µl of plasma eluted in a final volume of 15 µl, while Plasma/Serum Circulating and Exosomal RNA Purification Mini Kit (Norgen) was employed, according to the manufacturer’s instructions, to extract RNA from 1 ml of plasma eluted in a final volume of 100 µl. Only RNA samples extracted with the same method were pooled together for analysis.

### Nuclear and cytoplasmic RNA extraction

Cell fractionation to separate nucleus and cytoplasm was performed using the following protocol. Cells were incubated 10 min on ice in hypotonic buffer A (10 mM TRIS HCL ph 7.9, 10 mM KCl, 15 mM MgCl_2_, 200 U/ml RNaseOUT, Thermo Fisher Scientific) and lysed by adding 0.5% NP40. Nuclei were collected by centrifugation at 4000 rpm for 30 min at 4 °C. RNA from nuclear pellet and cytoplasmic supernatant was extracted with TRI Reagent Solution and TRIzol LS (Thermo Fisher Scientific), respectively, according to the manufacturer’s instructions. The efficiency of the separation of the two fractions was assessed by RNU48 positive control for the nuclear fraction.

### Reverse transcription and real-Time PCR

RNA was quantified using NanoDrop 2000 (Thermo Fisher Scientific) and, if not previously performed during the RNA extraction step, it was subjected to DNase I treatment (Roche). cDNA was then synthesized using the iScript cDNA synthesis kit (Bio-Rad) from normalized amounts of RNA (up to 1 µg RNA) in a final volume of 20 µl. For plasma-derived RNA, we retrotranscribed fixed RNA volumes: 5 µl (out of a total RNA volume of 15 µl) in case of RNA extracted with miRNeasy Serum/Plasma Advanced Kit (QIAGEN) and 15 µl (out of a total RNA volume of 100 µl) in case of RNA extracted with Plasma/Serum Circulating and Exosomal RNA Purification Mini Kit (Norgen). 3 µl of diluted cDNA (1:5) were then used to perform real-time PCR in a 10 μl reaction mix, with iQ SYBR Green (Bio-Rad). Thermal cycling conditions were the following: 95 °C for 3 min, then 40 cycles at 95 °C for 15 s and 60 °C for 30 s, followed by melting curve analysis. A no template control (NTC) was included in every assay. Primers are specified in Table S4. To rule out the possibility of primer dimers or non-specific amplification, we analyzed single peak of melting curves and we run PCR products on 3% agarose gels. Real-time PCR data were analyzed with the ΔΔCt method. The ΔCt value was determined by subtracting the Ct value of a reference gene (HUPO, GAPDH or HPRT) from the Ct value of the target gene. Relative expression was calculated with the 2^ − ΔΔCt method.

### Digital PCR

Circulating free DNA was isolated, amplified and analyzed for *MET* copy number variation (CNV) by droplet digital PCR (ddPCR) as previously described [47]. CircMET digital PCR (dPCR) was performed with a QuantStudio 3D Digital PCR System (Thermo Fisher Scientific) according to the manufacturer’s instruction. Briefly, 5 μl of cDNA (derived from plasma RNA extracted with Plasma/Serum Circulating and Exosomal RNA Purification Mini Kit, Norgen) were loaded onto each chip using a QuantStudio 3D digital PCR chip loader along with the recommended amount of QuantStudio 3D Digital PCR Master Mix v2 (Thermo Fisher Scientific) and of a FAM dye-labeled custom assay (specified in Table S4) designed on the circMET back-spliced junction target region (Bio-Rad). Amplification was carried out in a ProFlex 2X Flat PCR System (Thermo Fisher Scientific) at the following conditions: 96 °C × 10 min; 60 °C × 2 min, 98 °C × 30 s, 39 cycles; 60 °C × 2 min. Finally, chips were transferred into a QuantStudio 3D Digital PCR Instrument (Thermo Fisher Scientific) for imaging. Absolute quantification data, expressed as copies/μl of input cDNA, were elaborated through the QuantStudio 3D AnalysisSuite Cloud Software, followed by conversion of the results into copies/ml of plasma. Each sample was loaded and analyzed in duplicate, and a duplicate blank sample was included in each run.

### Absolute RNA quantification

Absolute RNA quantification was performed as described previously [48] with minor modifications. We first determined the amount of total RNA per cell. For this, the cell number (~ 1 × 10^6^ cells) was measured using a Countess cell counter (Thermo Fisher Scientific) and total RNA was extracted using Direct-zol RNA Miniprep (Zymo Research) with in-column DNase I treatment according to the manufacturer’s instructions. RNA was quantified using a NanoDrop 2000 (Thermo Fisher Scientific) and, under the assumption of negligible RNA loss, the mean total RNA per cell was estimated (7.72 ± 0.8 pg). Next, we generated standards for circMET and *MET.* Both targets were PCR amplified with PfuTurbo DNA Polymerase AD (Agilent), using primers that added a T7-promoter sequence. CircMET was amplified from a linear circMET construct that contains the circMET back-splicing junction. Linear circMET was PCR amplified from 100 ng of genomic DNA using PfuTurbo DNA Polymerase AD (Agilent). Oligonucleotides are specified in Table S4. The PCR product was cut with HindIII and XhoI restriction enzymes and cloned into the HindIII-XhoI sites of the pcDNA3 vector. *MET* was amplified from the full-size *MET* cDNA expressing vector [49, 50]. After purification, 1 µg of each PCR product was in vitro transcribed with T7 RNA polymerase (Roche) for 3 h at 37 °C, followed by DNase I treatment (Roche) for 15 min at 37 °C. RNA was then purified with RNeasy MinElute Cleanup Kit (QIAGEN), quantified with NanoDrop 2000 (Thermo Fisher Scientific) and checked on 1% denaturing agarose gel. The molecular weight of each RNA standard was calculated based on its sequence (396,516 g/mol for 1,235 nt circMET; 1,341,331.2 g/mol for 4,176 nt *MET*) and serial dilutions (10^11^ to 10^7^ copies) were performed in 1 mg/ml yeast tRNA (Thermo Fisher Scientific). To determine absolute amounts of circMET and *MET*, reverse transcription was performed with iScript cDNA synthesis kit (Bio-Rad) on each serial dilution of in vitro transcribed standard, alongside 1 µg of total RNA from cell lines. 3 µl of diluted cDNA (1:5) was then used to perform Real-time PCR with specific primers. For each RNA of interest, knowing its absolute amount per µg of total RNA, as well as the amount of total RNA per cell, we were able to calculate the number of molecules per cell. To evaluate the exon 2-skipped *MET* transcript, we subtracted canonical *MET* mRNA copy number (obtained using hMet conv ex2 primers designed on *MET* exon 2–3 junction) from that of total *MET* transcript (obtained using hMet ex14/15 primers designed on *MET* exon 14–15 junction).

### RNase R treatment

Total RNA (5 µg) was treated with 20 U RNase R (Epicentre) for 60 min at 37 °C. RNA was then purified with RNeasy MinElute Cleanup Kit (QIAGEN) and quantified with NanoDrop 2000 (Thermo Fisher Scientific). Concentration of MOCK control RNA was normalized to that of the RNase R-treated sample. cDNA was then synthesized using the iScript cDNA synthesis kit (Bio-Rad) and Real-time PCR was performed with iQ SYBR Green (Bio-Rad) as described above. Real-time PCR data were expressed as 2^ − ΔΔCt. For each gene, the ΔCt value was determined by subtracting the Ct value of the MOCK sample from the Ct value of the RNaseR-treated sample.

### Northern blot

DIG-labeled probe was produced by in vitro transcription with DIG-RNA labeling mix (Roche) of a 131 bp PCR template produced with the oligonucleotides hsa_circMET_0020882_JS3_FOR and 246_T7_REV (specified in Table S4). Transcription with T7 RNA polymerase (Roche) was carried out for 3 h at 37 °C, followed by DNase I treatment (Roche) for 15 min at 37 °C. RNA was then purified with RNA Clean and Concentrator-5 (Zymo Research), quantified with NanoDrop 2000 (Thermo Fisher Scientific) and checked on 1% denaturing agarose gel.

100 µg of GTL16 RNA were treated with 100 U RNase R (Epicentre) for 15 min at 37 °C. RNA was then purified with RNeasy MinElute Cleanup Kit (QIAGEN) and quantified with NanoDrop 2000 (Thermo Fisher Scientific). 7.5 µg of GTL16 RNA treated or not with RNase R were denatured with one volume of 2 × RNA Loading Dye (New England Biolabs, NEB) for 4 min at 70 °C followed by 2 min on ice and loaded on 1% denaturing agarose gel. Electrophoresis was carried out overnight at 15 V. RNA was transferred onto Hybond N + membrane (GE Healthcare) by capillarity overnight in 10X SSC and cross-linked with UV at 1200 Hz. Prehybridization and hybridization were performed in NorthernMax buffer (Ambion) at 68 °C (30 min and overnight respectively). 1 µg of DIG-labeled probe in 10 ml ULTRAhyb Buffer (Ambion) was used for hybridization. The membrane was then washed at hybridization temperature for 30 min with 1X SSC 0.1% SDS, then 0.1X SSC 0.1% SDS and finally 1X SSC. The membrane was finally processed for DIG detection (hybridization with anti-DIG antibody, washing and luminescence detection) with the DIG Wash and Block Buffer Set and the DIG luminescence detection kit (Roche), according to the manufacturer instructions.

### Genomic DNA extraction and GCN analysis

Genomic DNA was extracted using DNeasy Blood & Tissue Kit (QIAGEN), according to the manufacturer’s instructions. Real-time PCR was performed with 30 ng of DNA per single reaction using iQ SYBR Green (Bio-Rad) as described above. Primers designed to span centromeric regions were used to normalize data for aneuploidy. Analysis of resistant samples was normalized to the relative parental cells. Primers are specified in Table S4.

### CfDNA analysis

CfDNA was extracted from plasma using Maxwell® RSC ccfDNA plasma kit (Promega) according to the manufacturer’s instructions. Real-time PCR was performed with ®Easy EGFR kit (Diatech Pharmacogenetics). Real-time PCR data were analyzed by the ΔΔCt method. The ΔCt value was determined by subtracting the Ct value of the reference gene (EGFR control mix) from the Ct value of the target gene (T790M or Ex19del). Relative expression was calculated with the 2^ − ΔΔCt method. The NGS panel Guardant360 (Guardant Health, Inc.) was used for digital sequencing of cell-free circulating tumor DNA isolated from a blood draw.

### Western blot

Western blot assay was performed as described previously [51] with minor modifications: cells were harvested with RIPA Buffer (50 mM Tris HCl pH 8; 150 mM NaCl; 0.1% SDS; 0.5% sodium deoxycholate, 1% NP40; 1 mM Phenylmethanesulfonyl fluoride; 10 mM NaF; 1 mM Na3VO4, supplemented with protease inhibitor cocktail) and incubated 20 min on ice, centrifuged at 14,000 × g for 15 min at 4 °C. Protein lysates were loaded in NuPAGE Bis–Tris Protein Gels (Thermo Fisher Scientific) according to the manufacturer’s instructions. The following antibodies were used: mouse anti-Tubulin, Sigma-Aldrich Cat# T5201; Rabbit anti-Exportin-1/CRM1 (D6V7N), Cell Signaling Technology Cat# 46,249.

### Immunohistochemistry, immunofluorescence and fluorescent in situ hybridization

H&E and immunohistochemistry (IHC) on human samples were performed as routine clinical practice. The following antibodies were used: Rabbit anti-c-MET (SP44), Spring bioscience Cat# M3440; Rabbit anti- P-MET (D26), Cell Signaling Technology Cat# 3077. Pictures were taken with a BX51 microscope (Olympus). Fluorescent in situ hybridization (FISH) and assessment of MET/CEP7 ratio was performed as described previously [46] with ZytoLight ® SPEC MET/CEN 7 Dual Color Probe (ZytoVision GmbH). Pictures were taken with a BX61 fluorescence microscope (Olympus). FISH images were processed with Cytovision software. Murine H&E, IHC and human/mouse cellular immunofluorescence (IF) were performed as described previously [51, 52]. Antibodies for IHC: Mouse anti-c-MET (3D4), Thermo Fisher Scientific Cat# 18–7366; Rabbit anti-P-MET, Cell Signaling Technology Cat# 3126. Antibodies for IF: Mouse anti-MHC, Developmental Studies Hybridoma Bank Cat# MF20 (deposited to the DSHB by Fischman, D.A), Goat anti-Mouse IgG (H + L) Cross-Adsorbed Secondary Antibody Cyanine3, Thermo Fisher Scientific Cat# A10521. Fluorescent imaging was performed using a Leica TCS SP5 confocal system (Leica Microsystems).

### Padlock probes and rolling-circle amplification

CircMET detection with Padlock probes and Rolling Circle Amplification (RCA) was performed as described previously [53] with minor modifications. LNA primer, circMET padlock probe and CY3-labelled decorator probe sequences are specified in Table S4. Padlock probe was 5’ phosphorylated at a concentration of 10 μM with 0.2 U/μl T4 PNK (New England Biosciences, NEB) in 1 × PNK buffer A and 1 mM ATP (New England Biosciences, NEB) for 30 min at 37 °C, followed by 10 min at 65 °C. DEPC-H_2_O (Sigma-Aldrich) and RNase-free PBS (Sigma-Aldrich) were used during the entire procedure. 4 µm FFPE tissue sections were mounted on Superfrost Plus slides (Thermo Fisher Scientific) and dewaxed. Tissue was permeabilized with 0.1 mg/ml pepsin (Roche) in 0.1 M HCl at 37 °C for 30 min, washed in PBS and fixed with 3.7% formaldehyde for 10 min. Upon PBS washes, sections were dehydrated and air-dried. All the subsequent molecular in situ reactions were carried out in Secure-seals (Grace Bio-Labs) in 100 μl reaction volume and incubated in a humid chamber. The Secure-Seals were mounted over the tissue and the wells were rehydrated by a brief flush with PBS-0.05% Tween-20 (PBS-T). 1 μM of LNA primer was added to the slides with 20 U/μl of TRANSCRIPTME reverse transcriptase (Blirt), 500 μM dNTPs (Thermo Fisher Scientific), 0.2 μg/μl BSA (Thermo Fisher Scientific), and 0.8 U/μl RNaseOUT Recombinant Ribonuclease Inhibitor (Thermo Fisher Scientific) in the 1X reverse transcriptase reaction buffer. Slides were incubated for 3 h at 37 °C. After incubation, slides were washed briefly with PBS-T, followed by a postfixation step with 3.7% formaldehyde for 45 min at room temperature. After postfixation, samples were washed by flushing the Secure-seals chambers with PBS-T. Hybridization and ligation of the padlock probes were performed as follows. The reaction was carried out with 100 nM phosphorylated padlock probe in a mix of 1 U/μl TTh ligase (Blirt), 0.4 U/μl RNase H (New England Biosciences, NEB), 0.2 μg/μl BSA (Thermo Fisher Scientific), 0.05 mM KCl, 20% formamide in TTh ligase buffer. Incubation was performed first at 37 °C for 30 min, followed by 45 min at 45 °C. After ligation, slides were washed by flushing the chambers with PBS-T. RCA was performed with 1 U/μl phi29 DNA polymerase (New England Biosciences, NEB) in the supplied reaction buffer with 250 μM dNTPs (Thermo Fisher Scientific), 0.2 μg/μl BSA (Thermo Fisher Scientific), and 5% glycerol. Incubation was carried out for 5 h at 37 °C. After RCA, samples were washed by flushing the Secure-seals chambers with PBS-T. RCA products were visualized using 100 nM decoration probe in 2 × SSC (NaCl 0.3 M, trisodium citrate 30 mM, pH 7), 20% formamide, 30 ng/µl DAPI (Sigma-Aldrich) at 37 °C for 20 min. Slides were then washed again with PBS-T, Secure-seals were removed and slides were dehydrated. The dry slides were mounted with Fluoromount Aqueous Mounting Medium (Sigma-Aldrich). Fluorescent imaging was performed using a TCS SP5 confocal system (Leica Microsystems).

### Proliferation, anchorage-independent cell growth and drug sensitivity assays

Proliferation was evaluated by CellTiter-Glo (Promega) starting from 2 × 10^3^ HCC827 cells seeded on 96-well plates and incubated in the presence/absence of the indicated drugs for up to 7 days. Relative percentages were calculated by setting at 100% the average value of each curve on day 0. For anchorage-independent cell growth assay, cells were suspended in 0.45% type VII low-melting agarose (Sigma-Aldrich) in 10% FBS medium at a density of 6 × 10^4^ cells/well, plated on a layer of 0.9% agarose in 10% FBS medium in 6-well plates and cultured for two weeks at 37 °C with 5% CO_2_. Relative percentages were calculated by setting at 100% the average colony counts in DMSO. Images were acquired with a Leica DMIRE2 microscope (Leica Microsystems). For drug sensitivity (IC_50_) experiments, 2 × 10^3^ HCC827 cells were seeded on 96-well plates in the presence of serial dilutions of cetuximab, afatinib or erlotinib (Selleckchem). After incubation at 37 °C in 5% CO_2_, CellTiter-Glo (Promega) was used to measure luminescence according to the manufacturer’s protocol. Results were analyzed and plotted using GraphPad Prism version 9 software.

### siRNAs and transfection

siRNA XPO1 (Thermo Fisher Scientific) was used to down modulate Exportin-1. CY3 Negative control #1 siRNA (Thermo Fisher Scientific) was used as a negative control. Transfection was performed with Lipofectamine 2000 according to the manufacturer’s instructions.

### Bioinformatics analysis

For evaluation of circMET abundance and frequency, we analyzed expression data from publicly available databases [17, 54–58]. In particular, we plotted RNA-seq scores of both human (*n* = 9) and mouse (*n* = 6) MET circRNAs reported in circBase [54]. Corresponding circRNA sequences were downloaded to obtain molecular weight of expected amplicons. We expanded this analysis including all human MET circRNAs (*n* = 43) reported in circAtlas [58], with their corresponding conservation among vertebrates. The online tool http://bioinformatics.psb.ugent.be/webtools/Venn/ was used to create Venn diagrams of MET circRNAs detected from different databases. For the top 11 MET circRNA we also plotted RNA-seq mean expression and mean junction ratio, which was defined as the ratio between back-splicing junction reads and total number of reads aligned to the junction site [59]. Finally, we extended this analysis by downloading all circRNAs and parental gene expression in about 2000 cancer samples from the MiOncoCirc compendium (v0.1.release.txt and fpkm_matrix.csv from https://mioncocirc.github.io/download/ respectively) [17]. To evaluate the frequencies of circRNAs, we counted the number of tumors in which at least two reads of the circRNA junctions were present. After that, we measured the abundance of each circRNA from v0.1.release.txt by counting the total amount of reads among all tumor samples and ranking them. For the most abundant circRNAs (first quartile of the above-mentioned analysis), we calculated the Spearman correlation between circRNAs and the matched linear RNAs using the Python module scipy.stats.spearmanr. Concordance among different methodologies used to assess MET expression and activity status was graphically represented as a heatmap. Concordance of each comparison between two methods, expressed in terms of percentage, was calculated as the ratio between the number of samples resulting positive (or negative) by both methods and the sum of samples which were positive (or negative) by either one or both methods.

### Statistical analysis

Unless otherwise specified, results are expressed as mean ± SEM of at least two independent experiments (*n* = 2). Data were subjected to Student’s t-test (two-tailed, with *P* < 0.05 considered significant; ^NS^*P* > 0.05; *P < 0.05; **P < 0.01; ***P < 0.001; ****P < 0.0001). Additional statistical analysis (Pearson’s and Spearman’s correlation analyses) as well as specific number of repeats (*n*) are stated in the figure legends.

## Results

### CircMET RNA identification

**Fig. 1:**
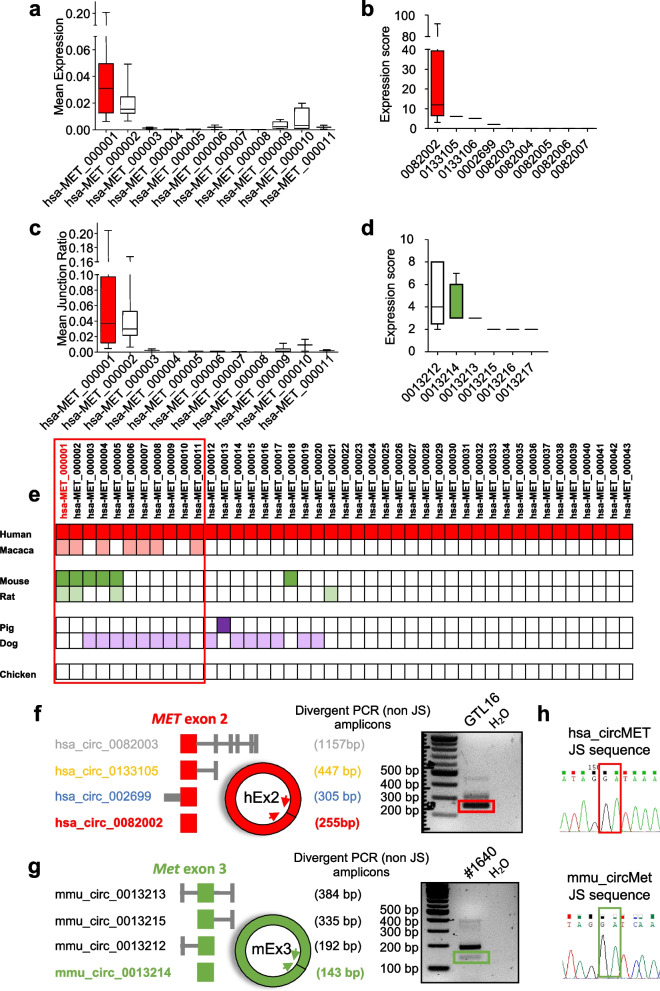
CircMET RNA identification. **a, b** Box plots of mean expression (**a**) and RNA-seq scores (**b**) of the indicated human MET circRNAs according to circAtlas [58] and circBase [54] dataset collections, respectively. CircMET is highlighted in red. **c** Mean junction ratio of MET circRNAs identified using circAtlas [58]. Mean junction ratio is defined as the ratio between back-splicing junction reads and the total number of reads aligned to the junction site. CircMET is highlighted in red. **d** RNA-seq scores of the indicated murine Met circRNAs according to circBase repository [54]. CircMet is highlighted in green. **e** Schematic representation of MET locus-derived circRNA conservation across vertebrates based on circAtlas [58] conservation analysis output. Filled boxes indicate the presence of the circRNA in each distinct species. Human circMET is reported in red, whereas murine circMET is reported in green. **f**,** g** Schematic representation of predicted MET locus-derived circRNA products containing human *MET* exon 2 (**f**) or mouse *Met* exon 3 (**g**) and related validation by PCR analysis in human gastric cancer (GTL16) and mouse sarcoma (#1640) cells, respectively. Intron–exon circRNA structure and predicted amplicon lengths are graphically represented on the left. Divergent non junction-spanning (non JS) primers were used to detect multiple exon 2- or exon 3-derived circRNAs. PCR bands corresponding to human and murine circMET are highlighted by a red and green box, respectively. **h** Sanger sequencing of human and murine PCR amplicons in the red and green boxes of panel f and g, respectively. Back-spliced junctions (JS) are highlighted

Technological advances in high-throughput sequencing and the development of innovative bioinformatics algorithms have allowed a systematic annotation of circRNAs in biological samples. Thereby, to identify circRNAs potentially encrypted in the *MET* locus, we interrogated several publicly available circRNA databases merging multiple RNA-seq datasets of circRNAs (Fig. S1a) [17, 54–58]. Interestingly, we found that *MET* exons generated multiple circular RNA isoforms (Fig. 1a, b), a process that is typically proportional to the number of encoded exons per gene [17]. By integrating different catalogs of circRNAs encoded by the *MET* locus we focused our attention on one circRNA (hsa-MET_000001 or hsa_circ_0082002), hereafter named circMET. Indeed, circMET was identified by all independent studies (Fig. S1a) and it stood out among other candidates also in terms of i) abundance (Fig. 1a, b), ii) circular/linear fraction (Fig. 1c), and iii) evolutionary conservation (Fig. 1d, e). We noticed that the most represented MET circRNAs, including circMET, originate from the longest exon of *MET* (human *MET* exon 2, homologous to the mouse *Met* exon 3; Fig. S1b). In late 1990s, M. Park’s group discovered an alternatively spliced *MET* transcript variant, highly expressed in tumor cells, lacking the ATG-containing exon 2 and failing to produce any detectable protein product in vivo [60]. Exon 2 skipping was proposed as a mechanism to decrease the abundance of the full-size *MET* mRNA, yet the fate of the excised exon 2 has never been investigated. Using divergent primers, we verified the expression of circMET in human and murine cell lines as well as the production of additional circRNAs originated from a process of circularization involving the inclusion of the adjacent exons (Fig. 1f, g and Fig. S1b). We applied Sanger sequencing on gel-extracted divergent PCR products to define the exact circMET back-spliced junction sequence. Our analysis perfectly matched the nucleotide string deposited in the interrogated circRNA databases (Fig. 1h), confirming the expected structure of circMET. Remarkably, only a fraction of circRNAs exhibits robustness in terms of annotation and abundance. This subtype is typically characterized by specific genomic features [17]. Accordingly, circMET is flanked by the longest introns of *MET* (Fig. S1b), which are enriched in multiple repetitive elements, a genomic architecture that actively favors the biogenesis of circRNAs [61], thus in part explaining circMET prevalence.

### CircMET RNA characterization

**Fig. 2:**
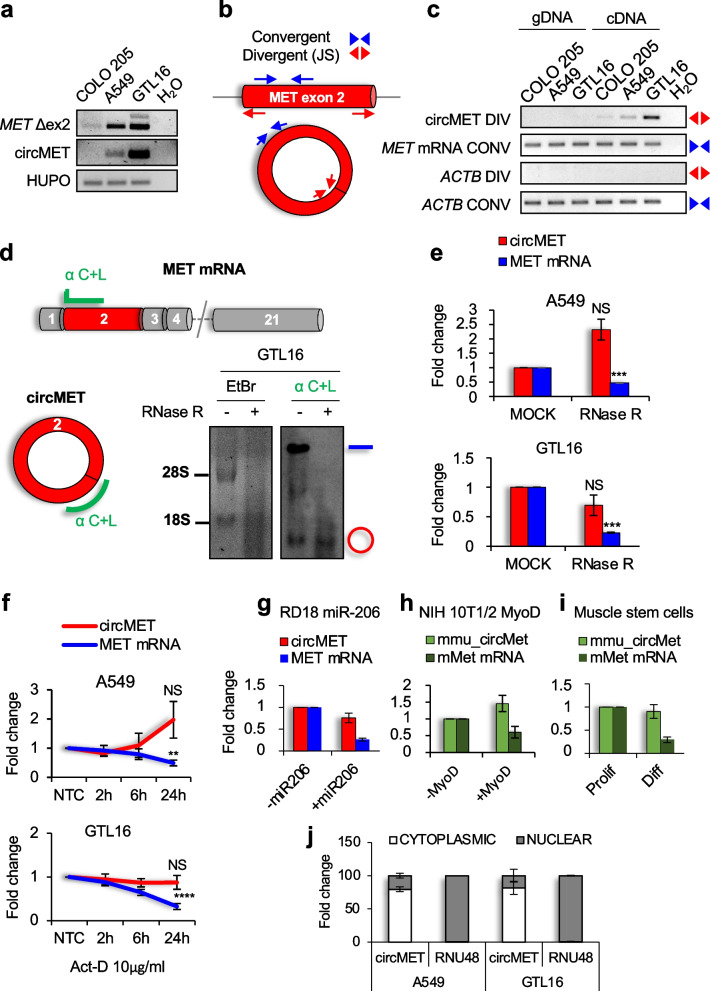
CircMET RNA characterization. **a** PCR analysis of the indicated transcripts. *MET* Δex2 represents the 7-Kb *MET* transcript lacking exon 2. **b** Schematics of primer pairs designed to amplify linear *MET* exon 2 (convergent) or its deriving circMET RNA product (divergent). **c** PCR analysis on genomic DNA (gDNA) and complementary DNA (cDNA) derived from the indicated cell lines. Junction-Spanning (JS) divergent primers were used to specifically detect circMET, while convergent primers were used to detect linear *MET* mRNAs. *ACTB* was used as a control. **d** Schematic representation of the probe used for Northern blot analysis together with its location on the linear and circular *MET* RNAs and Northern blot on 7.5 µg total RNA from GTL16 cell line treated or not with RNase R. The linear and the circular RNA forms are indicated next to the gels with the ‘‘–’’ and ‘‘o’’ symbols, respectively. **e** Real-time PCR analysis of linear MET and circMET levels upon RNase R digestion in the indicated cell lines. **f** Real-time PCR analysis of circMET turnover in the indicated cells treated with Actinomycin-D (*n* = 5). **g-i** Real-time PCR analysis of circMET and linear MET mRNA levels during myogenic differentiation of RD18 human rhabdomyosarcoma cells conditionally expressing miR-206 (**g**), NIH 10T1/2 murine fibroblasts conditionally expressing MyoD (**h**), and murine muscle stem cells in proliferation and differentiation medium (**i**). **j** Quantification of circMET levels in nuclear and cytoplasmic fractions of the indicated cell lines. RNU48 was used as a nuclear positive control. Data are expressed as mean ± SEM. ^NS^*P* > 0.05; **P < 0.01, ***P < 0.001, ****P < 0.0001, Student’s t test

In agreement with Park’s results [60], we detected exon 2-skipped *MET* RNA in all tested cell lines except for COLO 205 (Fig. 2a). Concordantly, also circMET was undetectable in this cell line, suggesting a functional correlation between *MET* exon 2 skipping and circMET formation. Furthermore, we validated the circMET head-to-tail junction by using circRNA-specific divergent primers (Fig. 2b). As expected, circMET was exclusively observed using cDNA as a template. In contrast, convergent primers recognized *MET* exon 2 in both genomic and cDNA templates (Fig. 2c). To verify circMET stability, we first treated total RNA with RNase R, an RNA exonuclease that specifically digests linear RNA molecules, regardless of their intrinsic structures, preserving both circRNAs and lariats, and we designed a Northern Blot probe to expressly detect both linear and circular MET transcripts. While RNase R strongly affected *MET* mRNA abundance, circMET exhibited resistance to exonuclease degradation (Fig. 2d). In addition, the higher circMET resistance to exonuclease activity was also confirmed by real-time PCR assay (Fig. 2e). Moreover, we analyzed endogenous circMET turn-over by using Actinomycin D to inhibit transcription in a time-course assay. Even in this context, circMET exhibited a higher half-life than linear MET mRNA (Fig. 2f). Since MET downregulation is physiologically requested to promote muscle differentiation, we evaluated circMET stability in different models of myogenesis (Fig. S2a, b, c). We have previously demonstrated that microRNA-206 reactivates terminal myogenic differentiation in rhabdomyosarcoma cells by targeting the 3’UTR of the MET receptor [52]. By using a rhabdomyosarcoma cell line that conditionally expresses miR-206, we verified that miR-206 induction promoted *MET* mRNA downregulation, but spared circMET (Fig. 2g). Indeed, circMET originates from *MET* exon 2, thus escaping from microRNA regulation, which solely involves the 3’UTR of target genes. We confirmed this observation by forcing myogenic conversion of murine fibroblasts using the master transcriptional factor MyoD1 (Fig. 2h and Fig. S2b) and by promoting terminal differentiation of muscle stem cells in vitro (Fig. 2i and Fig. S2c) Taken together, these results indicate that circMET is an abundant, stable and conserved RNA molecule, which originates from the back-splicing of *MET* exon 2. CircRNAs are generally located in the cytosol, where they modulate gene function by: i) titrating out microRNAs from their natural mRNA targets, ii) interacting with different RNA binding proteins or iii) translating novel protein products [19]. This particular subcellular localization favors circRNA extracellular release and delivery as both free circulating RNA molecules and exosomes-encapsulated circRNAs, thus supporting their potential use as biomarkers [62]. We verified that also circMET was particularly enriched in the cytosol (Fig. 2j), and its localization was mainly driven by the Exportin-1 nuclear shuttle, as demonstrated by the inhibition of circMET cytoplasmatic enrichment upon Exportin-1 silencing (Fig. S2d, e).

### CircMET epitomizes the MET status in cancer

**Fig. 3:**
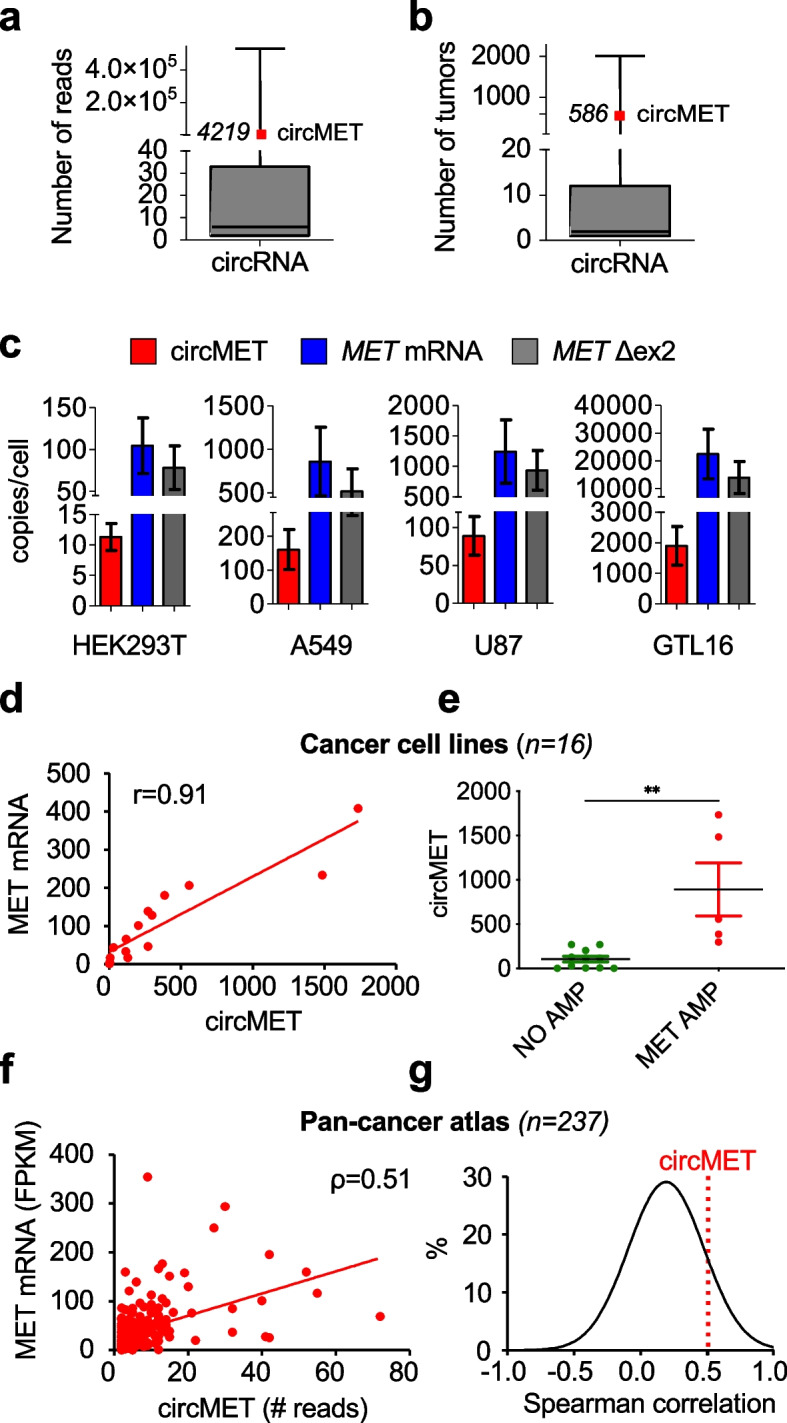
Assessment of circMET expression in cancer cells and primary tumors. **a**,** b** Box plots of abundance (number of reads) (**a**) and frequency (number of tumors) (**b**) of all circRNAs detected in tumor samples based on MiOncoCirc compendium-based analysis [17]. cicMET is indicated in red, along with its corresponding values. **c** Quantitative real-time PCR analysis of circMET, linear *MET,* and *MET* transcript lacking exon 2 (*MET* Δex2) in the indicated cell lines. **d** Correlation between circMET and *MET* linear mRNA levels measured by real-time PCR in cancer cell lines (*r* = Pearson correlation coefficient) (*n* = 16). **e** Dot plot of circMET levels measured by real-time PCR in cancer cell lines with and without *MET* amplification (*n* = 16). **f** Correlation between circMET and linear *MET* mRNA in tumor samples (ρ = Spearman correlation coefficient) (*n* = 237) based on MiOncoCirc data [17]. **g** Spearman rank correlation of the most abundant circRNAs (1^st^ quartile of the data included in panel** b**). CircMET coefficient is indicated with a dotted red line (*ρ* = 0.51). Data are expressed as mean ± SEM. **P < 0.01, Student’s t test

MET is a clinically relevant cancer-related target but the determination of *MET* amplification and overexpression remains challenging [41]. We hypothesized that thanks to its intrinsically high abundance and stability, circMET could be exploited as a novel biomarker capable to mirror the MET status in cancer cells. We therefore interrogated a compendium of thousands of circRNAs identified by exome sequencing on tumor tissues [17]. Remarkably, circMET emerged among the most abundant and frequently annotated cancer-associated circRNAs (Fig. 3a, b) [17]. Furthermore, we performed an accurate quantitation of circMET in several cell lines characterized by different levels of *MET* mRNA. Interestingly, the absolute value of circMET ranged from about 10 copies/cell in HEK293T (very low *MET* expressors), to around 2000 copies/cell in GLT16 (a *MET-*amplified cell line) (Fig. 3c), where MET also acts as an oncogenic driver. Given the limitations of the amplicon-based NGS approaches to assess gene copy number (GCN) variations, we verified whether circRNA-based quantitation could provide an alternative effective approach. Generally, expression of the cognate linear RNA is not considered a reliable parameter to evaluate circRNA abundance and vice versa [17, 63]. Notwithstanding, specific circRNAs have been correlated to gene amplification in tumors and thus proposed as potential surrogate markers [17]. To test if circMET belonged to this rare category, we initially evaluated circMET levels in 16 different cancer cell lines (Table S1). Intriguingly, our analysis revealed a strong positive association between circMET and *MET* mRNA expression (Pearson correlation coefficient *r* = 0.91) (Fig. 3d) especially in *MET*-amplified cells (Fig. 3e). We extended our observation by reanalyzing the circRNA landscape of cancer, where circRNAs are found generally down-modulated and weakly correlated with parental gene expression [17]. Also in this comprehensive database, we observed a robust correlation between circMET and linear *MET* RNA (Fig. 3f, g).

### In vitro identification of MET-driven acquired resistance and molecular therapy response using circMET

**Fig. 4:**
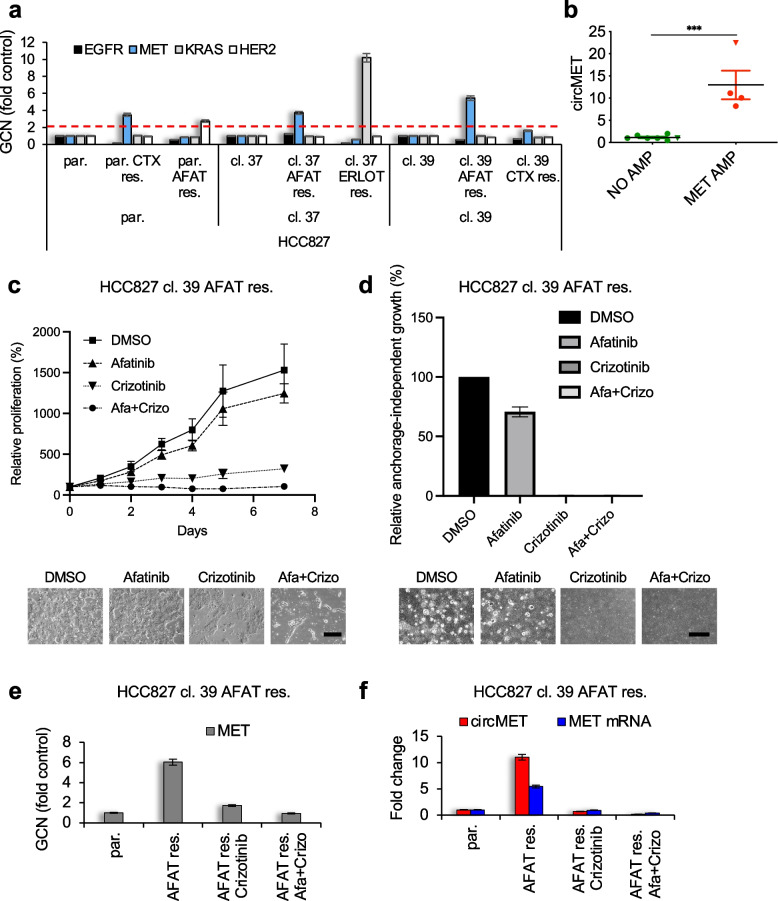
CircMET evaluation reveals MET-driven acquired resistance and mirrors MET-targeted therapy response in vitro. **a** Gene Copy Number (GCN) analysis of the indicated parental (par.) and clonal (cl.), sensitive and resistant (res.) HCC827 cell populations. Red dashed line indicates the twofold threshold for *MET* amplification. **b** Dot plot of circMET levels measured by real-time PCR in 11 among HCC827 and WiDr parental and resistant cells with and without *MET* amplification. Triangles are referred to WiDr parental and resistant cells. **c** Proliferation assay of afatinib-resistant HCC827 clonal cells treated with the indicated inhibitors. Representative pictures are shown below (scale bar = 500 μm). **d** Soft-agar colony formation assay of afatinib-resistant HCC827 clonal cells treated with the indicated inhibitors. Representative pictures are shown below (scale bar = 500 μm). **e**,** f** *MET* GCN (**e**) and real-time PCR analyses (**f**) of afatinib-resistant HCC827 cell subclones upon 7 days of treatment with the indicated inhibitors. AFAT, afatinib; ERLOT, erlotinib; CTX, cetuximab. Data are expressed as mean ± SEM. ***P < 0.001, Student’s t test

Since the identification of optimal biomarkers to select patients who could benefit from MET-targeted therapies is still challenging [64], we explored whether a circRNA-based quantitation could provide an alternative effective approach. Thereby, we analyzed circMET levels in two distinct in vitro models of acquired resistance, a lung *EGFR*-mutant (HCC827) (Fig. S3a, b, c) and a colon *BRAF*-mutant (WiDr) cell line [44]. Across the 7 different resistant cell lines (parental and clones) analyzed, circMET signal was strongly enriched only in the 4 *MET*-amplified cell lines, specifically 3 HCC827- and 1 WiDr-derived resistant populations (Fig. 4a, b and Fig. S3d, e, f). Finally, we explored the possible use of circMET to dynamically track the response to MET-directed therapies. *MET*-amplified HCC827 cells resistant to the pan-HER inhibitor afatinib (cl. 39 AFAT res.; Fig. 4a and Fig. S3b) were treated with the clinically approved dual MET and ALK inhibitor crizotinib. This treatment greatly interfered with cell proliferation and anchorage-independent growth (Fig. 4c, d) and the rare surviving cells, negative for *MET* amplification (Fig. 4e) progressively lost both *MET* and circMET expression (Fig. 4f). Interestingly, a parallel subclone with a lower degree of *MET* amplification (cl. 37 AFAT res.; Fig. 4a and Fig. S3b) did not show sensitivity to crizotinib treatment alone, but displayed a remarkable growth reduction when MET inhibition was combined with anti-HER treatment (Fig. S4a). Accordingly, in this clone exhibiting HER/MET codependency, *MET* GCN as well as *MET* and circMET expression were only affected in the combination regimen (Fig. S4b, c).

### CircMET detection in the plasma of cancer patients and correlation with the MET status of the related tissue biopsies

**Fig. 5:**
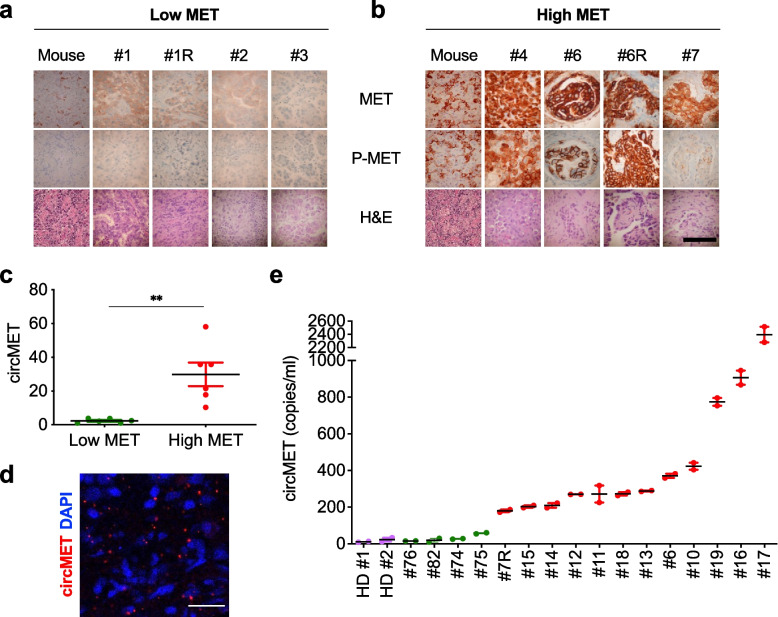
Circulating circMET in cancer patients correlates with the MET status of the corresponding tissue biopsies. **a**,** b** Immunohistochemical analysis of low- (**a**) and high- (**b**) MET-expressing human lung adenocarcinomas and mouse rhabdomyosarcoma (Mouse) specimens with the indicated antibodies (scale bar = 100 μm). **c** Dot plot of circMET levels measured by real-time PCR in FFPE tumor samples (*n* = 12). Samples included in the analysis are listed in Table S2. **P < 0.01, Student’s t test. Data are expressed as mean ± SEM. **d** In situ detection of circMET in a *MET*-amplified FFPE tumor sample (case #4) using padlock probes and rolling circle amplification (scale bar = 25 μm). **e** Absolute quantification of circMET levels measured by digital PCR in the indicated liquid biopsies of cancer patients (*n* = 16) and healthy donors (HD) (*n* = 2). Each sample was analyzed in duplicate: both circMET values are shown for each patient (colored dots), along with their average value (black lines) and SEM. Low and high circMET expressors are indicated in violet/green and red, respectively. Clinical information of the primary human samples analyzed is provided in Table S2

To translate our findings from cell line models to patients, we analyzed circMET in formalin-fixed paraffin-embedded (FFPE) tumors, including both HGF-driven murine sarcomas [43] and human lung and colon adenocarcinomas (ADK) (Table S2). Given that the parameters used for FISH scoring of *MET* amplification are still debatable [41, 64], we initially assessed the *MET* status in a subset of these tumors by MET and phospho-MET immunohistochemistry (IHC) and classified them as either low or high MET expressors (Low MET – High MET, Fig. 5a, b and Table S2). Analysis of FFPE samples confirmed that circMET was upregulated in high-MET tumors (Fig. 5c, d and Fig. S5a, b). Thereby, we hypothesized that assessment of circMET could be exploited to evaluate the status of MET in a non-invasive manner. FISH and/or IHC analyses to score MET activity were used to select patients from a retrospective cohort where matching plasma samples were available as well (Table S2). Remarkably, plasma levels of circulating circMET reflected the MET status in the corresponding tissue samples (Fig. 5e, Table S2 and Fig. S5a, b). Overall, our analysis suggested that circMET detection in liquid biopsy, which co-occurs with more traditional MET activity markers (Fig. S5a, b), could be applied as a complementary strategy to better implement patient stratification, especially in cases of i) ambiguous FISH, ii) uncertain IHC, iii) lack of available tissue, or iv) equivocal NGS output.

### Non-invasive tracking of MET-driven acquired resistance and monitoring of therapy response using a circMET-based detection strategy

**Fig. 6:**
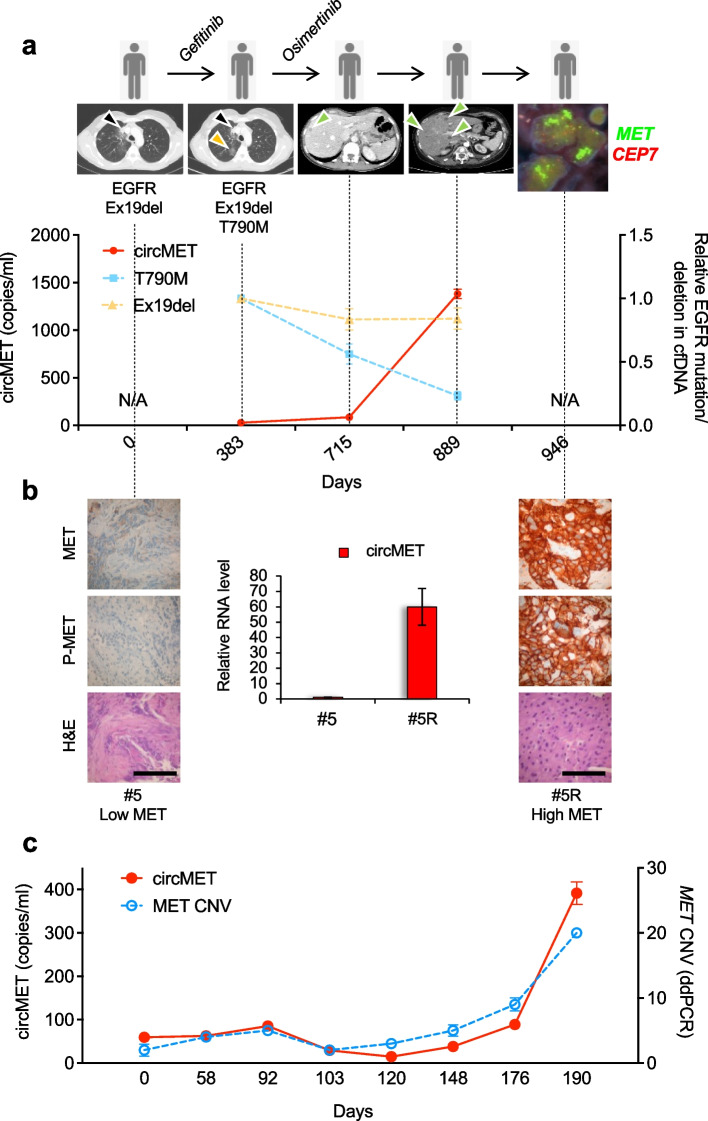
CircMET allows non-invasive tracking of MET-driven acquired resistance and therapy response in cancer patients. **a** Upper panel: clinical history of lung adenocarcinoma case #5. Black and yellow arrows on CT scans indicate primary and secondary lung lesions, respectively, while green arrows indicate liver lesions. FISH for *MET* in the post-therapy biopsy (case #5R) is shown on the right. *MET* is labeled in green and centromeres in red. Lower panel: dPCR on circMET along with real-time PCR on *EGFR* T790M and *EGFR* Ex19del upon cell-free DNA (cfDNA) extraction from liquid biopsies longitudinally obtained at the indicated time points (N/A = not available). **b** Immunohistochemical and real-time PCR analyses on FFPE biopsies collected before and after therapy (case #5 and case #5R) (scale bar = 100 μm). **c** dPCR analysis of circMET upon cfRNA extraction along with ddPCR-based Copy Number Variation (CNV) assessment on cfDNA from liquid biopsies obtained at the indicated time points of a previously described colorectal cancer case [44, 47]. Data are expressed as mean ± SEM

Molecular cancer therapies frequently result in patient relapse for the rapid emergence of resistant clones, a condition prevalently associated to the rate of tumor heterogeneity at the genomic and functional level [65]. Recent evidence support a superior value of liquid biopsy rather than more traditional and invasive tissue biopsies to investigate cancer heterogeneity and clonal evolution during treatment [66]. Moreover, *MET* amplification is emerging as a common mechanism of acquired resistance [38], providing the rationale for the use of MET-directed therapies in selected patients. Considering the limitations and the cost of ctDNA NGS approaches, we monitored free circMET in the plasma of an EGFR-mutated advanced lung ADK patient (case #5 in Table S2) under treatment with a first-generation tyrosine kinase inhibitor (Fig. 6a). Unfortunately, the patient experienced progressive disease in the lung and distant secondary lesions, due to the acquisition of the T790M *EGFR* mutation. Accordingly, a second line treatment with osimertinib was initiated. Once again, the disease progressed in the liver. Cell-free DNA (cfDNA) analysis was performed to identify new genetic lesions potentially involved in osimertinib resistance (Table S3). Concomitantly, we tested our circMET-based detection approach. Both cfDNA and cfRNA methods detected the emergence of a *MET*-amplified clone, tracked over time by circMET monitoring (Fig. 6a). In parallel, cfDNA analysis of the *EGFR* status revealed the stable presence of *EGFR* exon 19 deletion and a decrease in T790M *EGFR* mutation. Moreover, a liver biopsy was obtained to investigate genetic aberrations involved in the metastatic lesion (case #5R in Table S2). IHC and circMET quantification from FFPE tissues classified the metastasis as high MET (Fig. 6b), and FISH analysis confirmed the genetic amplification of the *MET* gene (Fig. 6a). Finally, the performance of the circMET-based detection strategy was retrospectively assessed in a metastatic *BRAF* V600E-positive rectal cancer case. CircMET levels dynamically tracked *MET* amplification in response to crizotinib [44], and the evolution of *MET* hyper-amplification as an additional mechanism of acquired resistance [47] (Fig. 6c). Altogether, these results underpin the use of circMET as a novel potential biomarker to track *MET* amplification and to evaluate response to anti-MET directed therapies.

## Discussion

Tissue biopsy in advanced cancer faces the critical issue of anatomical localization and tumor accessibility, as in the case of metastatic lung cancer that frequently colonizes the central nervous system as well as the mid-lung or retroperitoneum. Thus, tumor biopsy can be extremely challenging and is not always feasible in the clinical practice for the associated risks. Conversely, liquid biopsy is a minimally invasive procedure that offers the opportunity to monitor tumor burden and evolution over time [67]. Moreover, the superior value of this approach is now emerging in comprehensively profiling tumor heterogeneity and dynamically tracking clonal evolution and genomic architecture during treatment [66]. Most studies are currently focused on ctDNA analysis, however, increasing evidence shows that several non-coding RNAs (ncRNAs) involved in tumorigenesis represent potential biomarkers for cancer diagnosis [68]. Only recently, the implications of ncRNA, including circRNA, have become evident in clinical oncology [69]. Since our genome is pervasively transcribed, giving rise to multiple ncRNA species, and since cancer cells are proficient in releasing ncRNA species in the blood circulation, the growing interest for the use of ncRNA molecules in the clinical practice is not surprising. In the last decade, microRNAs have been extensively investigated in tumor profiling and patient stratification, also in the context of lung cancer, including blood-based assays [70]. However, microRNAs are not useful to directly track coding gene abundance since they are exclusively non-coding species. As biomarkers, circRNAs are particularly attractive for their stability and the possibility to monitor also parental gene expression. CircRNAs can freely circulate in peripheral whole blood [23], in extracellular vesicles [71] and in urine [17], thus representing novel potential biomarkers in liquid biopsy analyses. Generally, the expression of circRNAs is finely regulated and it is uncoupled from that of their host linear mRNAs [72]. In contrast, circMET showed a strong positive correlation with MET levels in both tumor cell lines and primary specimens, suggesting its potential application for tracking the status of the *MET* gene. Accordingly, both in vitro models of MET-driven acquired resistance and analysis of primary tumor specimens supported the ability of circMET to discriminate tumor cells and patient samples characterized by high MET expression and phosphorylation. Moreover, we showed concordance between high MET activity in tissue biopsies and circMET in the blood of the same patients. Overall, both tissue and circulating circMET levels were consistent with the presence of high-MET activity indicators in the matched tumors, such as pMET positivity, which is not solely associated with MET GCN gain but also to its overexpression [41, 42]. In this scenario, circMET may represent an alternative circulating readout of MET activation. Despite the intrinsic limitations of our analysis, based on retrospective samples and therefore characterized by heterogeneous clinical annotation and limited material availability to systematically compare FFPE specimens and plasma from the same patients, it allowed us to verify circMET biomarker potential in parallel with more traditional analytical tools. Notably, we observed a dynamic positive correlation between circMET and *MET* gene GCN in plasma samples longitudinally collected from patients exhibiting *MET* amplification as a mechanism of drug resistance. Finally, circMET levels well correlated with the response to anti-MET molecular therapies. Remarkably, the possibility to apply latest generation nanopore sequencing in the liquid biopsy context could represent a sensitive and cost-effective strategy to gather genomic and transcriptional information from plasma specimens. Indeed, besides being applicable for *MET* GCN monitoring on cfDNA in lung cancer [73], nanopore technology has been recently successfully employed for detailed profiling of circRNAs as well [74, 75], opening new perspectives for the use of circRNAs as cancer biomarkers.

## Conclusions

Although this study was conducted on a limited number of patients, we provide a proof of concept that in the uncertain cases, where tissue biopsies are ambiguous or not available, the detection of circMET in a blood-based assay is a feasible and cost-effective approach to evaluate MET activity. While further investigations are still required to validate our findings in a larger cohort of patients, our data suggest that a circMET-based detection strategy represents a potential complementary liquid biopsy approach to assist decision-making for treatment in precision oncology.

### Supplementary Information

**Additional file 1:** **Fig. S1. **CircMET RNA *in silico* analysis. **Fig. S2. **circMET expression and cellular localization. **Fig. S3. **Generation and characterization of HCC827 and WiDr drug-resistant subpopulations. **Fig.S4. **CircMET evaluation reveals HER/MET codependency and mirrors combination therapy response *in vitro*. **Fig.S5. **Heatmap of concordance among assays applied to the primary samples. **Table S1. **Cancer cell lines assessed for MET and circMET expression, with indication of *MET* amplification status. **Table S2. **Clinical information of the primary samples included in the study. **Table S3. **Summary of the somatic alterations detected with Guardant360 Biopsy-Free Tumor Sequencing in case #5 upon relapse to osimertinib. **Table S4. **List of primers and probes.

## Data Availability

The datasets analyzed in the current study are available from the following public repositories: circBase: http://www.circbase.org [54], circRNADb: http://reprod.njmu.edu.cn/cgi-bin/circrnadb/circRNADb.php [55], deepbase v2.0: http://biocenter.sysu.edu.cn/deepBase/ [56], circpedia V2: http://yang-laboratory.com/circpedia/ [57], circAtlas: http://circatlas.biols.ac.cn [58], MiOncoCirc: https://mioncocirc.github.io [17]

## Abbreviations

circMET: Circular MET
circRNA: Circular RNA
cfDNA: Cell-free DNA
cfRNA: Cell-free RNA
ccMET: Circulating circMET
ctDNA: Circulating tumor DNA
FISH: Fluorescence in situ hybridization
IHC: Immunohistochemistry
IF: Immunofluorescence
FFPE: Formalin-fixed paraffin-embedded
dPCR: Digital PCR
ddPCR: Droplet digital PCR
GCN: Gene copy number
RCA: Rolling circle amplification
