# Supplementary material for: Exploring circular MET RNA as a potential biomarker in tumors exhibiting high MET activity
Source: J Exp Clin Cancer Res. 2023 May 12;42:120. doi: 10.1186/s13046-023-02690-5 (PMC10176894; doi:10.1186/s13046-023-02690-5)
Supplement: Supplementary file 1 — Additional file 1: Fig. S1. CircMET RNA in silico analysis. Fig. S2. circMET expression and cellular localization. Fig. S3. Generation and characterization of HCC827 and WiDr drug-resistant subpopulations. Fig.S4. CircMET evaluation reveals HER/MET codependency and mirrors combination therapy response in vitro. Fig.S5. Heatmap of concordance among assays applied to the primary samples. Table S1. Cancer cell lines assessed for MET and circMET expression, with indication of MET amplification status. Table S2. Clinical information of the primary samples included in the study. Table S3. Summary of the somatic alterations detected with Guardant360 Biopsy-Free Tumor Sequencing in case #5 upon relapse to osimertinib. Table S4. List of primers and probes. [file 13046_2023_2690_MOESM1_ESM.docx]

**ADDITIONAL FILE 1**

**SUPPLEMENTARY FIGURES**

**
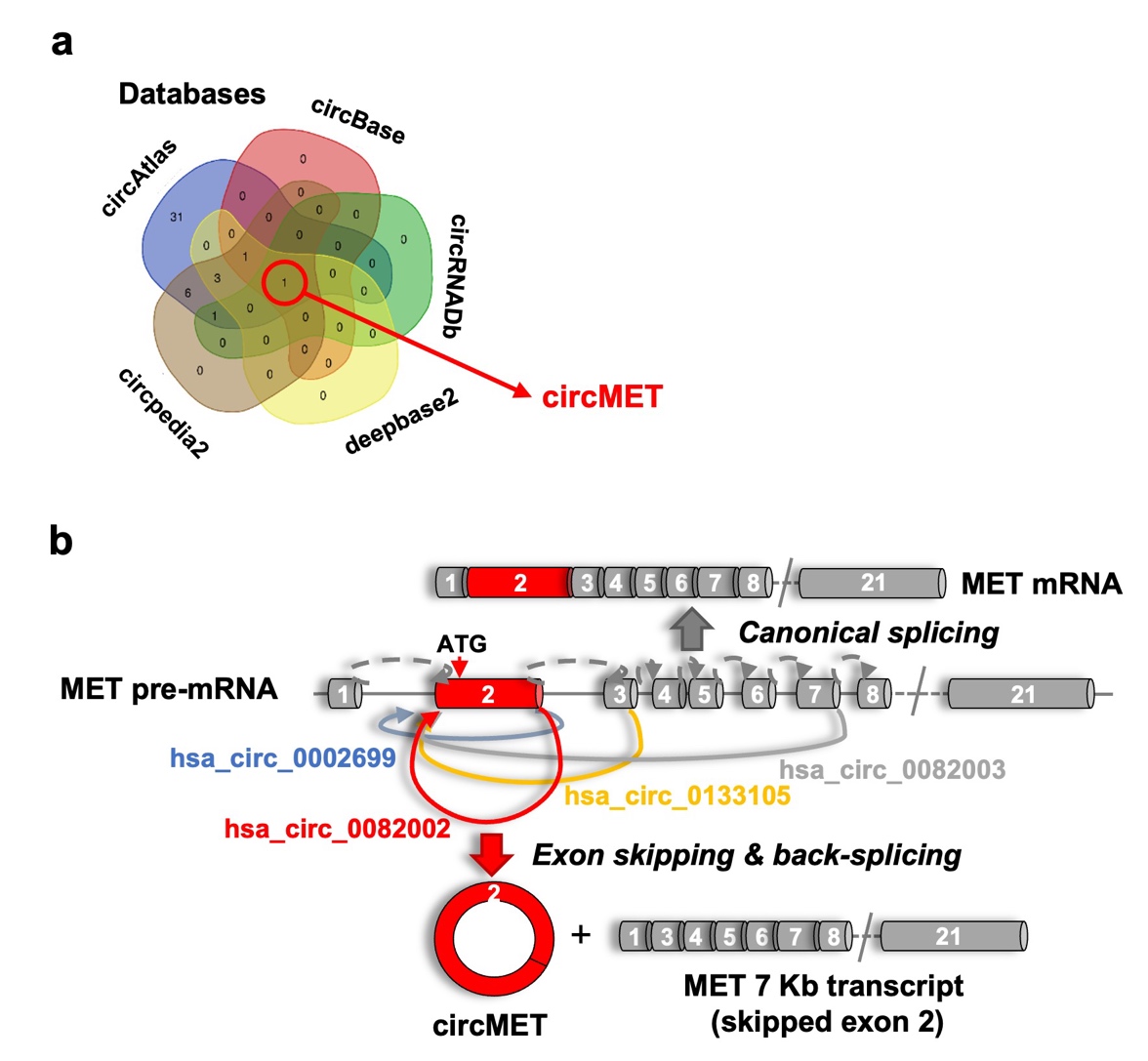
**

**Fig. S1.** CircMET RNA *in silico* analysis.

**a** Venn diagrams with the number of MET-derived circRNAs detected using the indicated databases. CircMET is circled in red. **b** Schematic representation of splicing events involving *MET* pre-mRNA (in the middle), which generate canonical *MET* mRNA (above), circRNAs and transcripts with skipped exons (below). Hemi-circle arrows indicate *MET* exon 2-containing circRNAs.

**
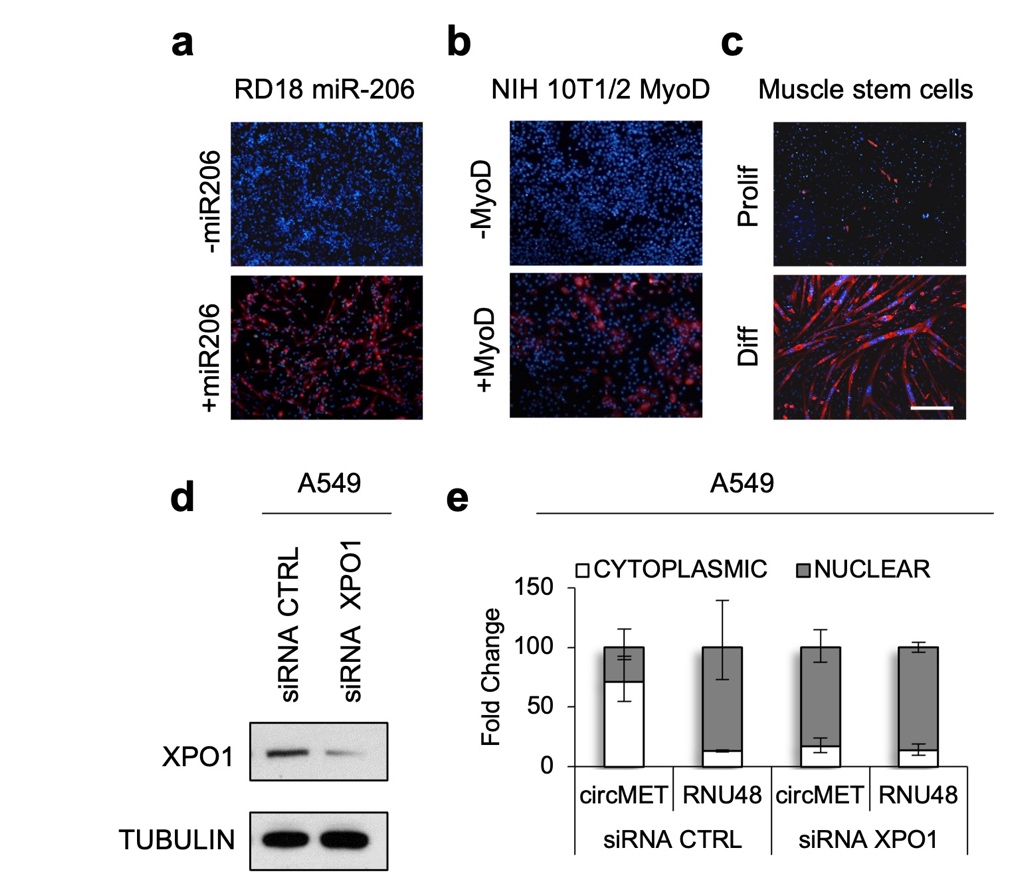
**

**Fig. S2.** circMET expression and cellular localization.

**a-c** Representative DAPI (blue) and MHC (red) immunofluorescence stainings of cellular systems used to assess circMET expression, including myogenic differentiation of RD18 human rhabdomyosarcoma cells conditionally expressing miR-206 (**a**), murine NIH 10T1/2 fibroblasts conditionally expressing MyoD (**b**) and murine muscle stem cells upon differentiation (**c**). Scale bar = 250 μm. **d** Western blot analysis of A549 cells transfected with XPO1 and Control siRNA. Tubulin was used as a loading control. **e** Quantification of circMET levels in nuclear and cytoplasmic fractions of A549 cells upon XPO1 silencing by siRNA and related control. RNU48 was used as a nuclear positive control. Data are expressed as mean ± SEM.


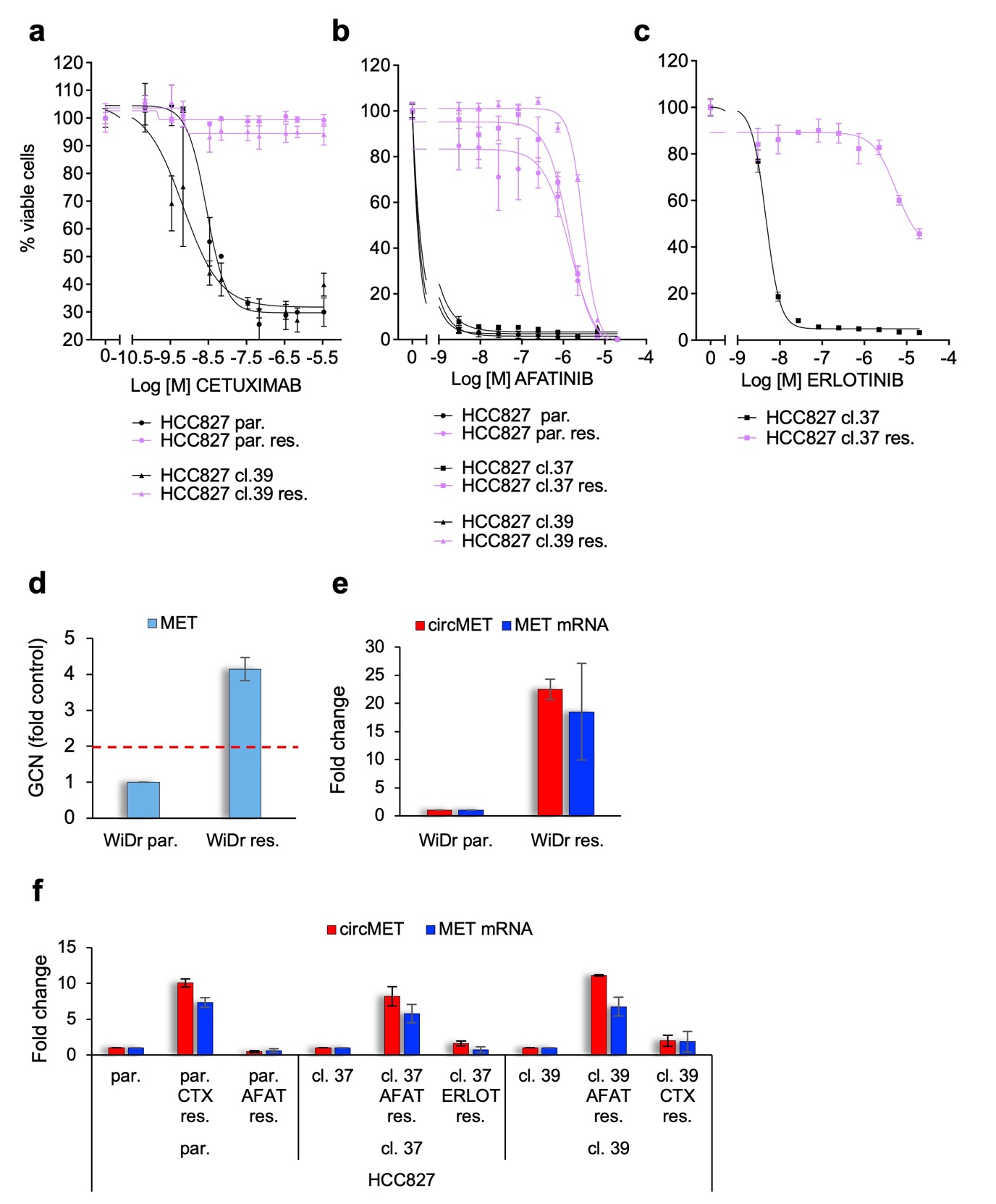


**Fig. S3.** Generation and characterization of HCC827 and WiDr drug-resistant subpopulations.

**a-c** Drug sensitivity (IC_50_) analysis of the indicated parental (par.) and clonal (cl.), sensitive and resistant (res.) HCC827 cell populations exposed to cetuximab (**a**), afatinib (**b**) and erlotinib (**c**), respectively. Data are expressed as mean ± SD. **d** Gene Copy Number (GCN) analysis of the indicated parental (par.) and resistant (res.) WiDr cell populations. Red dashed line indicates the 2-fold threshold for *MET* amplification. **e-f** Real-time PCR analysis of circMET and linear MET mRNA levels in parental (par.), clonal (cl.), sensitive and resistant (res.) WiDr (**e**) and HCC827 (**f**) cell populations. Data are expressed as mean ± SEM.

**
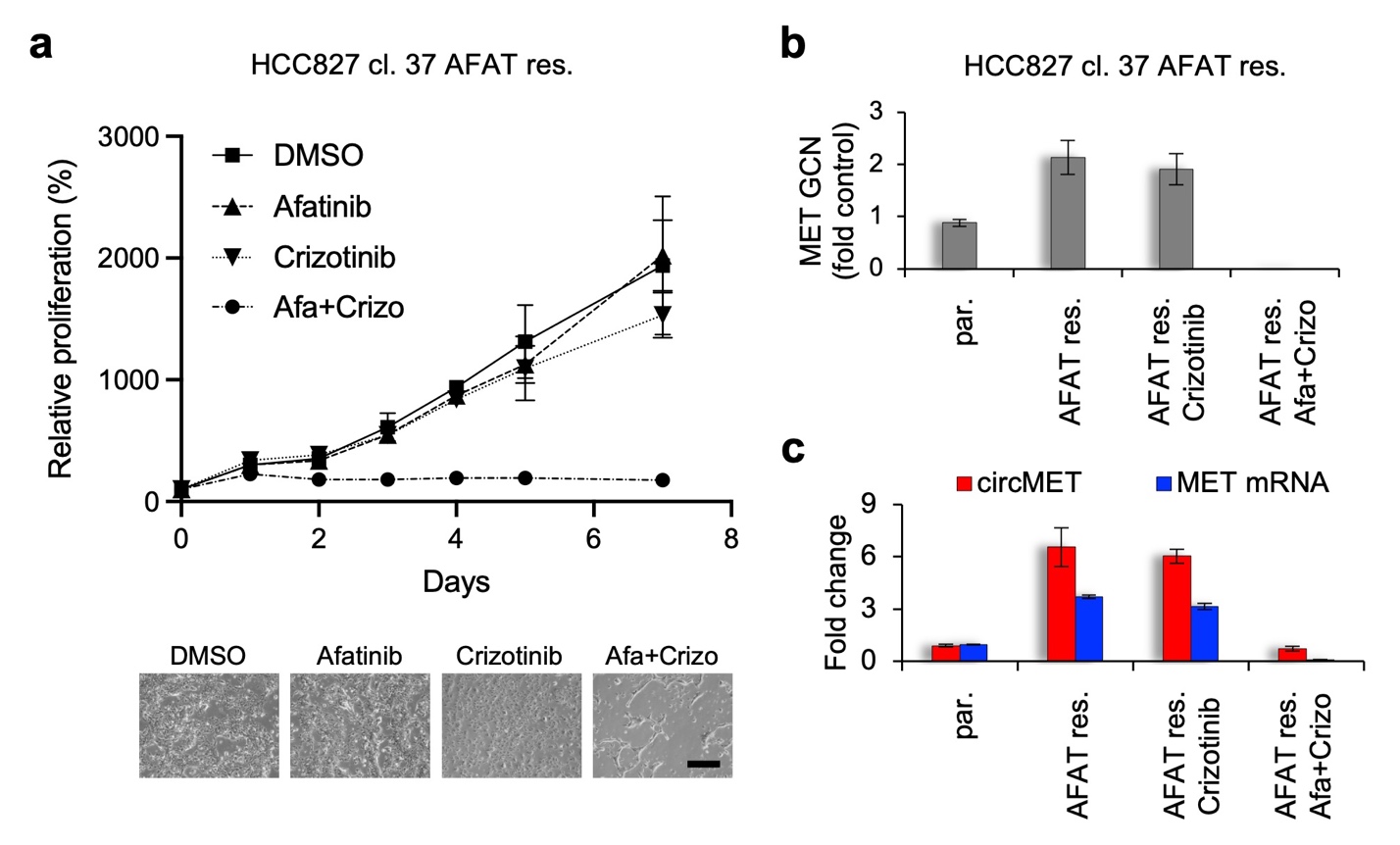
**

**Fig. S4.** CircMET evaluation reveals HER/MET codependency and mirrors combination therapy response *in vitro*.

**a** Proliferation assay of afatinib-resistant HCC827 clone 37 cells treated with the indicated inhibitors. Representative pictures are shown below (scale bar = 500 μm). **b-c** *MET* GCN (**b**) and real-time PCR analyses (**c**) of afatinib-resistant HCC827 clone 37 cells upon 7 days of treatment with the indicated inhibitors. AFAT, afatinib. Data are expressed as mean ± SEM.


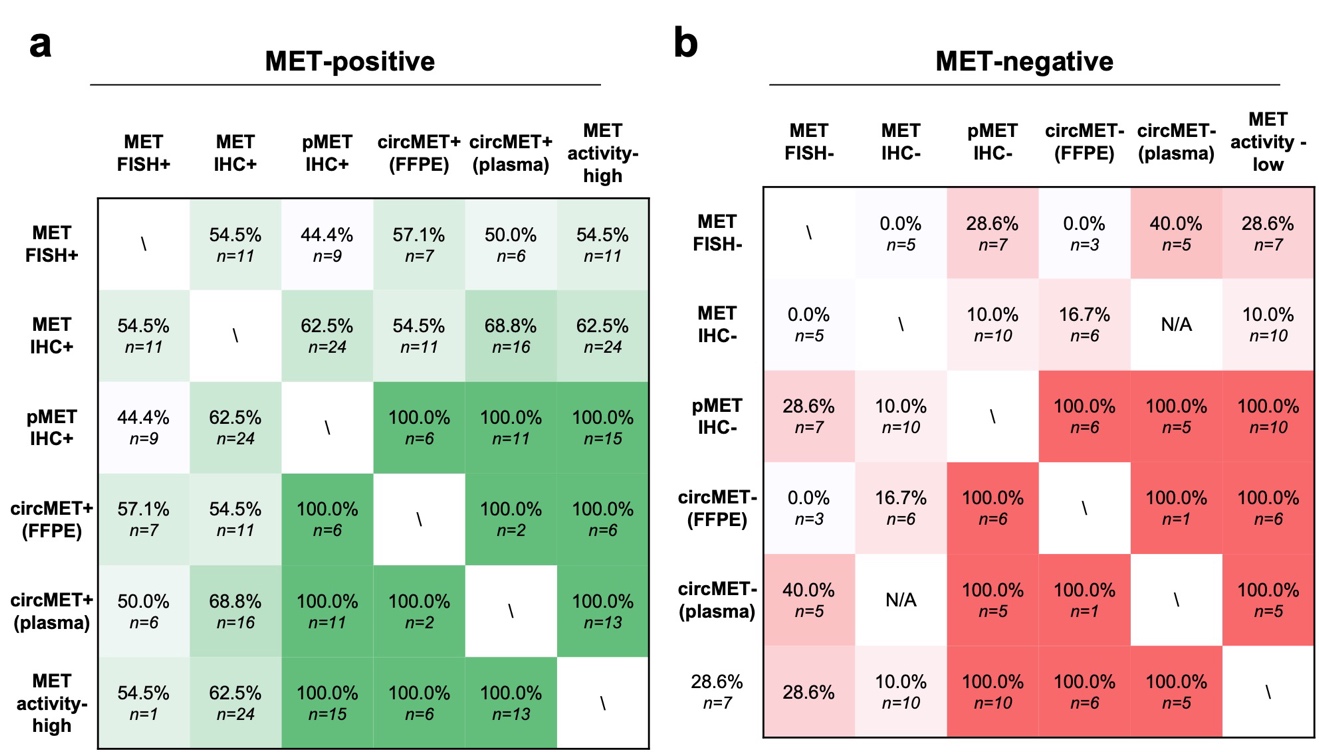


**Fig. S5.** Heatmap of concordance between assays applied to the primary samples.

Comparison of either MET-positive (**a**) or MET-negative (**b**) ‘calls’ among all the applied techniques. The indicated percentage at each intersection between a raw and a column refers to the fraction of dual positive (**a**) or negative (**b**) samples divided by the sum of the dual positive (or negative) plus the single positive (or negative) samples with either assay. The sample size *n* is shown for each pair of parameters. Increasing concordance is represented by lighter-to-darker shades of green (for MET-positive calls) or red (for MET-negative calls).

**SUPPLEMENTARY TABLES**

**Table S1.** Cancer cell lines assessed for MET and circMET expression, with indication of *MET* amplification status.

**Table S2.** Clinical information of the primary samples included in the study.

**Table S3.** Summary of the somatic alterations detected with Guardant360 Biopsy-Free Tumor Sequencing in case #5 upon relapse to osimertinib.

**Table S4.** List of primers and probes.
